# Predictive value of CHA2DS2-VASc score in radial artery occlusion after transradial coronary angiography

**DOI:** 10.3389/fcvm.2023.1157087

**Published:** 2023-06-12

**Authors:** Aydın Nadir, Nuray Kahraman AY

**Affiliations:** Faculty of Medicine, Bezmialem Vakıf University, Istanbul, Türkiye

**Keywords:** CHA2DS2-VASc score, radial artery, coronary angiography, trombose, antiagregantes

## Abstract

**Background:**

Radial artery occlusion is the most common complication of transradial catheterization. RAO is characterized by thrombus formation due to catheterization and endothelial damage. CHA2DS2-VASc scores are the current scoring systems used to determine the risk of thromboembolism in patients with atrial fibrillation. The aim of this study was to investigate the relationship of CHA2DS2-VASc score with radial artery occlusion.

**Methods:**

This prospectively designed study was included 500 consecutive patients who underwent coronary artery transradial catheterization for diagnostic or interventional procedures. The diagnosis of radial artery occlusion was made by palpation examination and Doppler ultrasound at the twenty-fourth hour after the procedure. Independent predictors of radial artery occlusion were determined by logistic regression analysis.

**Results:**

Radial artery occlusion was observed at a rate of 9%. The CHA2DS2-VASc score was higher in the group of the patients who developed radial artery occlusion (*p* < 0.001). Arterial spasm (OR: 2.76, 95% CI 1.18–6.45, *p*: 0.01), catheterization time (OR: 1.03, 95% CI 1.005–1.057, *p*: 0.01) and CHA2DS2-VASc score ≥ 3 (OR: 1.44, 95% CI 1.17–1.78, *p*: 0.00) as significant independent predictors of radial artery occlusion. A high CHA2DS2-VASc score was associated with the continuity of the occlusion after the treatment (OR:1.37, 95% CI 1.01–1.85, *p*: 0.03).

**Conclusions:**

An easily applicable CHA2DS2-VASc score of ≥3 has a predictive value for radial artery occlusion.

## Introduction

Coronary angiography (CAG) is still the gold standard diagnostic method for coronary artery occlusion. Transradial catheterization (TRC) is widely preferred in coronary angiography due to early mobilization, discharge, cost and patient comfort (1). The most common complications of transradial coronary angiography are hematoma at the intervention site, pseudoaneurysm in the radial artery, spasm and occlusion (2, 3). Although the rate of radial artery occlusion (RAO) varies from center to center, it is observed between 5% and 30%, usually asymptomatic (4). Some patients may experience pain, paresthesia, or decreased arm function at the site of the occlusion. Cases of arm ischemia have also been reported due to inadequate post RAO collateral circulation (5, 6). Although RAO is usually asymptomatic, radial artery patency is significant because it can be used as access in recurrent percutaneous coronary interventions (PCI), as a graft in coronary bypass surgery, or to create fistulas in hemodialysis patients.

RAO is characterized by damage to the endothelium due to catheterization and thrombus formation due to reduced blood flow (4). CHA2DS2-VASc scores are the current scoring systems used to determine the risk of thromboembolism in patients with atrial fibrillation (AF). In the studies, it has been reported that the CHA2DS2-VASc score provides information about the prognosis of various cardiovascular diseases regardless of the presence of atrial fibrillation (7, 8). The predictive value of the CHA2DS2-VASc score in the development of RAO following TRC is unknown. The main purpose of this study was to investigate the relationship between development of RAO after TRC and CHA2DS2-VASc score.

## Methods

This study was performed at high-volume cardiac center with the approval of the ethics committee dated 14 December 2021 and numbered 2021/387. The study included 500 patients who underwent radial coronary angiography in a single center between December 2021 and June 2022.

The patients with acute coronary syndrome (ACS) and stable angina who applied to the clinic with the indication of CAG were included in the study. Electrocardiogram (ECG), complete blood count, blood glucose, blood urea nitrogen (BUN), creatinine, serum electrolytes and cardiac enzyme (troponin, CK-MB) values were evaluated as recommended for routine clinical evaluation in coronary artery disease guidelines. Those with chest pain, dynamic ST-T wave changes on ECG, and cardiac enzyme levels more than five times at presentation were considered ACS. The patients with metabolic imbalance, undergoing chemotherapy for cancer, chronic kidney disease, and bleeding disorders were excluded from the study. Left radial artery was used for TRC. The patients who could not pass the Allen test and digital pulse oximetry test were excluded from the study. Patients who passed both tests underwent CAG through TRC and were followed up. 24 h after the catheter was removed from the radial artery, the radial artery flow pattern was examined by pulse examination and doppler ultrasonography(DUSG). The absence of a pulse on palpation in the radial artery and the absence of antegrade flow signal on DUSG were considered as RAO. The patients with radial artery occlusion were treated with subcutaneous low molecular weight heparin (LMWH) for 14 days in the morning and evening. The antiaggregant treatments they were using or started with the coronary procedure were also maintained. After two weeks of LMWH treatment, the radial artery was evaluated with DUSG. In the following period, antiaggregant (acetylsalicylic acid 100 mg, clopidogrel 75 mg) was administered to all the patients who developed RAO, whether recanalized or not, for 6 months.

The patients were divided into two groups as those who developed radial occlusion (RAO) after TRC and those who did not (nonRAO). CHA2DS2-VASc scores of all the patients were calculated. The basic clinical and demographic characteristics and the findings in the angiography laboratory were compared between the two groups. A logistic regression model was created to evaluate the relationship between female sex, hypertension, diabetes mellitus, hyperlipidemia, smoker, arterial spasm, catheterization time, CHA2DS2-VASc score variables and RAO. The relationship between CHA2DS2-VASc scores and post-treatment radial artery patency rates was evaluated by Cox regression analysis.

### Transradial angiography

We used standard transradial access. Local anesthesia was applied to the area to be intervened by placing the left hand in the extension and external rotation position. 0.5 cc Prilocaine (CITANEST® 2%) was used subcutaneously for local analgesics. Then, intra-arterial puncture was performed with a 20 G puncture needle. Following arterial pulsation, 45 mm 0.025'‘ non-flonized wire and a non-resistive 6F 15 cm radial sheath (TERUMO 6 F) were placed over the wire. Prior to diagnostic CAG, 100 mcg perlinganite and 5,000 IU unfractionated heparin were diluted in the sheath. The patients with radial spasm during the procedure were excluded from the study. A 0.035 inch diameter J-tipped wire arc from a 6F radial sheath was inserted into the aorta, then 6F metronic brand JL 3,5-4-4,5 and JR 4 coronary diagnostic catheters were used for diagnostic CAG. Antiaggregant was not administered to the patients with normal coronary arteries during CAG. Heparin was completed with 100 IU/kg in the patients with stenosis greater than 70% and stent surgery planned in the same session. Following CAG, sheath was removed and a TR band (Terumo, Somerset, NJ, USA) radial artery compression device was used to control bleeding. We used patent hemostasis and confirmed with oximetry probe. Average TR band compression for two hours. According to the guidelines, dual antiaggregant (ASA + clopidogrel or ticagrelor + ASA) was administered to the patients treated with ACS and only ASA to the patients with non-critical coronary plaques.

### CHA2DS2-VASc score

CHA2DS2-VASc score is the current scoring system used to determine the risk of thromboembolism in patients with AF. The CAHDS-VASc score, a scoring system used to predict atheroembolic events in patients with AF, includes parameters such as age between 65 and 74 years, female sex, heart failure, hypertension, diabetes, stroke, peripheral vascular disease. In the CHA2DS2-VASc scoring system, C (CongestiveHeartFailure) (1 point), H(Hypertension) (1 point), Age over 75 (2 points), Diabetes Mellitus (1 point), Stroke (2 points), Vascular disease(1 point), Age 65–75 age (1 point), Sex category (Famele 1 point) parameters are evaluated. In the nine-point scoring system, 0 = Low risk, 1 = Moderate risk, 2 and above = High stroke risk. The 2010 ESC guidelines that introduced the CHADS 2 score and the modified CHA2DS2 -VASc score for more detailed risk assessment, recommend oral anticoagulation for patients with a score >1 (8).

### Statistical analysis

SPSS statistical software pack (SPSS 26.0 for Windows, Inc., Chicago, IL, USA) was used for data analysis. In addition to descriptive statistics (mean, standard deviation), for the comparison of quantitative data Student's *t*-test was used for parameters with normal distribution and Mann-Whitney *U* test for parameters without normal distribution. Fisher's exact test and chi-square test were used for the comparison of qualitative data. A logistic regression model was used to identify the independent predictors of RAO. A cox regression model was used to determine the effect of CHA2DS2-VASc score on RAO. A *p* value of less than 0.05 was considered significant. Receiver-operating characteristic (ROC) curves were obtained for PLR to explore the sensitivity and specificity. ROC curve analysis was used to determine the optimum cutoff levels of CHA2DS2-VASc score to predict the occurrence of RAO.

## Results

**Figure 1:**
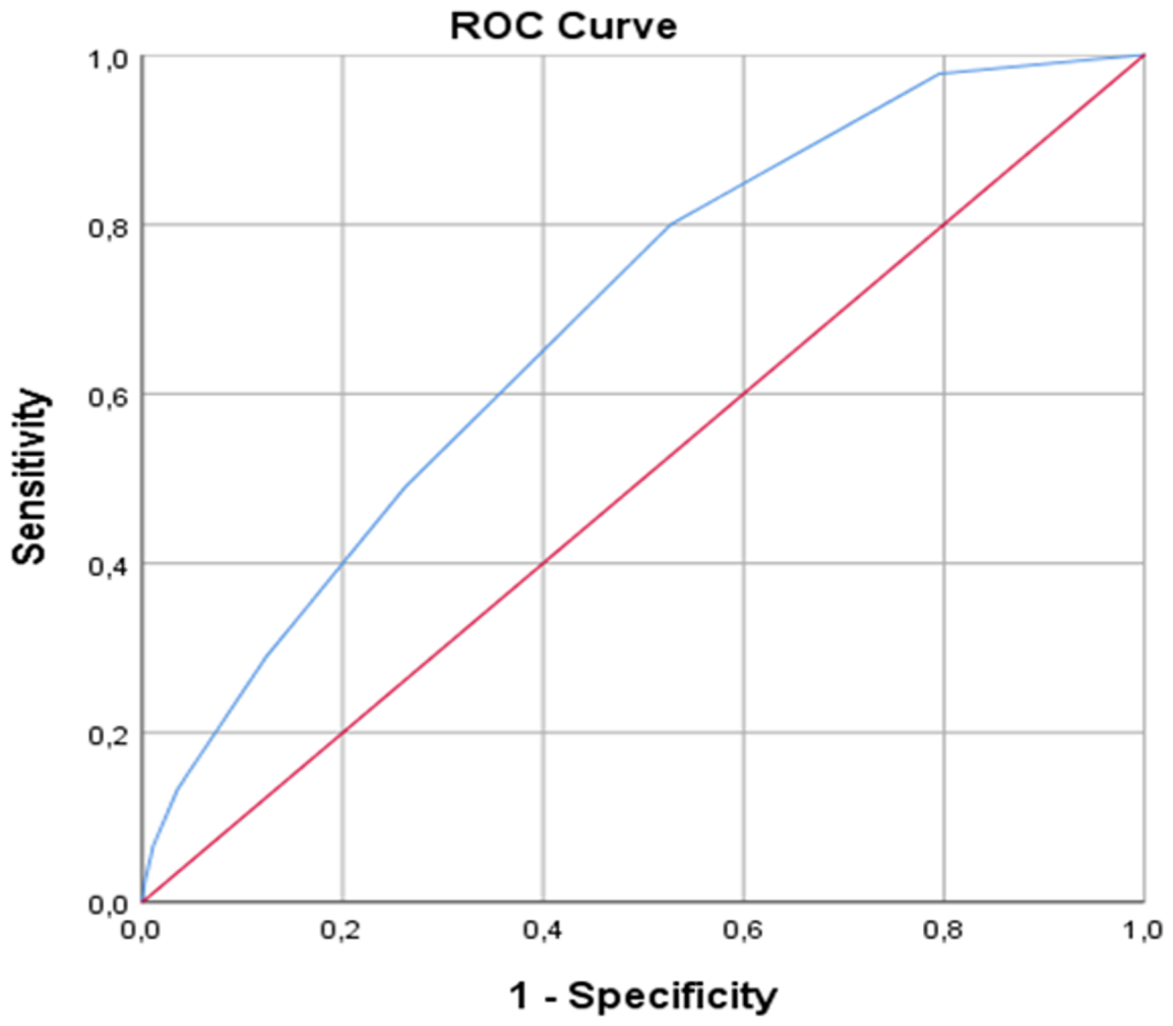
ROC curve analysis for cutoff value of CHA2DS2-VASc score for detecting RAO. ROC, receiver operating characteristic.

The rate of RAO development was 9% in 45 patients. While the use of atrial fibrillation, anticoagulant and antiaggregant was significantly higher in the RAO group, the proportion of the patients who did not receive any medical treatment was higher in the nonRAO group (*p* < 0.05). The mean CHA2DS2-VASc score was significantly higher in RAO group compared to nonRAO group (1.75 ± 1.3 vs. 2.77 ± 1.5, respectively, *P* < 0.001). There was no difference between the groups in terms of other demographic data (Table 1). The rate of arterial spasm development during the procedure was higher in the RAO group (*p* < 0.001). There was no difference between the two groups in terms of the rates of emergency or elective interventional or diagnostic procedures and the mean procedure times (*p* > 0.05) (Table 2). When the patients were classified according to the CHA2DS2-VASc score, the majority (71%) were found to have a score of 0–2. RAO was observed at a higher rate in patients with a high CHA2DS2-VASc score. The area under the ROC curve (95% confidenceinterval) for CHA2DS2-VASc score, as a predictor of RAO, was 0.687 (0.612–0.762) (*p* < 0.001). Using a cutpoint of ≥3, the CHA2DS2-VASc score correlated with the incidence of RAO with a sensitivity of 48.9% and specificity of 73.8% (Figure 1).

**Table 1:** Basic clinical and demographic characteristics of patients at the time of admission.

**Variables**	**nonRAO (*n* = 455)**	**RAO (*n* = 45)**	***p***
Age	62 ± 12.64	65 ± 12.7	0.532
Gender Male, (*n*, %)	206 (45.3)	11 (24.4)	0.071
Hypertansion (*n*, %)	286 (62.9)	28 (62.2)	0.526
Hyperlipidemia (*n*, %)	109 (24.1)	17 (37.8)	0.931
Diabetes (*n*, %)	117 (25.7)	16 (35.6)	0.155
Smoker (*n*, %)	119 (41.8)	22 (48.9)	0.428
Family history (*n*, %)	94 (29.7)	6 (13.3)	0.242
BMI	25.3 (±3.6)	23.8 (±2.8)	0.713
*AF*	*47* (10.3)	1 (2.2)	**0**.**003**
*Mean CHA2DS2-VASc score*	*1.75 *±* 1.39*	2.77 ± 1.55	**0**.**000**
Medication (*n*, %)	391 (85.9)	26 (57.7)	**0**.**000**
*Antiaggregant and anticoagulant*	*2* (0.4)	–	**0**.**00**
*ASA*	*112* (24.6)	8 (17.8)	0.307
*Clopidogrel*	*14* (3.1)	–	<0.001
*ASA and Clopidogrel*	*160* (35.2)	15 (33.3)	0.829
*ASA and Ticagrelor*	*58* (12.7)	2 (4.4)	**0**.**024**
*Warfarin*	*18* (4.0)	–	<0.001
*Rivaroxoban*	*27* (5.9)	1 (2.2)	0.143
Without medication (*n*, %)	64 (14.1)	19 (42.2)	**0**.**001**

RAO, radial artery occlusion; BMI, body mass index; ECG, elctrocardiogram; AF,atrial fibrilllation;

ASA, asetil salicylclic acid.

**Table 2:** Demographic characteristics of patients during angiography laboratory.

** **	**nonRAO (*n* = 455)**	**RAO (*n* = 45)**	***p***
**Coronaryangiography (*n*, %)**			
*Electively*	295 (64,8)	38 (84,4)	
Diagnostics	261 (57.4)	32 (71.1)	0.936
Interventional	34 (7.5)	6 (13,3)	0.559
*Urgent*	160 (35.2)	7 (15,6)	0,025
Diagnostics	30 (6.6)	0 (0,0)	**<0.001**
Interventional	130 (28.6)	7 (15,6)	0.689
Systolic blood pressure(mmHg)	135 ± 17.01	131 ± 15,36	0.125
Diastolic plood pressure(mmHg)	74,6 ± 8.31	73,8 ± 5,4	0.174
Heart rate/min	75,1 ± 10.8	69,2 ± 9,05	0.027
Procedure time/min	19.4 ± 9.1	15,3 ± 8,29	0.268
Arterial spasm			
Heparin dose/IU	4,456 ± 2,729	4,008 ± 2,521	0.358
TR band follow time/min	131 ± 18	128 ± 21	0.182

In Table 3, the incidence of RAO and post-treatment recanalization rates according to the CHA2DS2-VASc score are shown. Within two weeks of DMHA treatment, the radial artery recanalization rate was 66.6%. In the Cox regression analysis, it was determined that the high CHA2DS2-VASc score in the patients with RAO was significantly associated with the persistence of arterial occlusion after two weeks of treatment (OR:1.37, 95% CI 1.01–1.85, p:0.03).

**Table 3:** Radial artery occlusion and posttreatment recanalization rates according to CHA2DS2-VASc score.

**CHA2D2s-VASc score**	**Patients** **(*n*:500)**	**Radial artery occlusion (*n*, %)**	**Successful Radial Flow After LMWH (*n*, %)**
0	94 (18)	1 (1.1)	1 (100)
1	130 (26)	8 (6.2)	7 (87.5)
2	135 (27)	14 (10.4)	10 (71.4)
3	72 (14.4)	9 (12.5)	6 (66.6)
4	47 (9.4)	7 (14.5)	4 (57.1)
5	14 (2.8)	3 (21.4)	1 (33.3)
6	6 (1.2)	2 (33.3)	1 (50)
7	2 (0.4)	1 (50)	0 (0)

Univariate and multivariate analysis identified arterial spasm (OR: 2.76, 95% CI 1.18–6.45, *p*: 0.01), catheterization time(OR: 1.03, 95% CI 1.005–1.057, *p*: 0.01) and CHA2DS2-VASc score (OR: 1.44, 95% CI 1.17–1.78, *p*: 0.00) as significant independent predictors of RAO (Table 4).

**Table 4:** Multivariable logistic regression analysis showing the independent predictors of in radial artery occlusion.

Variables	Univariate		Multivariate	
	OR (95% CI)	*p*	OR (95% CI)	*p*
Female	0.95 (0.51–1.76)	0.88		
Hypertension	1.71 (0.86–3.41)	0.12		** **
Diabetes mellitus	1.57 (0.82–3.0)	0.16		
Hyperlipidemia	1.43 (0.73–2.79)	0.29		
Smoker	1.82 (0.98–3.38)	**0**.**05**	1.77 (0.92–3.41)	0.08
Arterial spasm	3.90 (1.77–8.62)	**0**.**001**	2.76 (1.18–6.45)	**0**.**01**
Catheterization time >15 min	1.03 (1.01–1.05)	**0.0 1**	1.03 (1.01–1.05)	**0**.**01**
CHA2DS2-VASc	1.55 (1.27–1.89)	**<0**.**001**	1.44 (1.17–1.78)	**<0**.**001**

## Discussion

In this study, we found that arterial spasm, catheterization time and high CHA2DS2-VASc score were predictive values in the development of RAO following TRC. Moreover, we found that the high CHA2DS2-VASc score was significantly associated with the persistence of arterial occlusion at the end of the two-week treatment following RAO.

The most common complication of TRC is RAO with a rate of 2%–18% (9). In the study, we detected 9% of RAO. Similar restenosis results were seen in many studies, in our study it was considered as subtotal occlusion in the case. Therefore, the rate of radial artery occlusion may be high. A thrombotic phenomenon is thought to be the basis for the formation of RAO. Placing sheath in the radial artery creates a thrombotic environment by causing local endothelial damage, intimal rupture, medial dissections, and interruption of blood flow in the artery. Besides, the compression applied after the procedure results in hemostasis, forming a nidus for thrombus formation (10). The CHA2DS2-VASc score is the current scoring system used to determine the risk of thromboembolism in patients with AF. In this study, we aimed to investigate the relationship between RAO, which is a thrombotic event, and CHA2DS2-VASc score. This is the first study in the literature to evaluate the relationship between CHA2DS2-VASc score in the development of RAO.

Studies have shown that factors such as female sex, diabetes, lower BMI, radial artery diameter ≤2.2 mm and radial artery-to-sheath ratio (AS ratio) < 1 have a predictive role in the development of RAO (11, 12). Female sex, low BMI and presence of diabetes expose a risk for RAO as they are associated with smaller artery size (11). Additionally, the higher incidence of RAO in women has been associated with their greater sensitivity to vascular spasm (13). Since an A/S ratio >1 is a risk for RAO, the use of small sheaths is recommended in diagnostic angiography and uncomplicated coronary interventions, especially in female patients. Furthermore, repeated insertion of the guidewire and angiographic catheters may trigger spasm, increasing the risk of RAO (14). Since we used 6F sheath in all the patients, we did not evaluate sheath size, but we determined that arterial spasm had a predictive value for RAO (OR: 2.76, 95% CI 1.18–6.45, *p*: 0.01). Apart from these, it is stated that the prolongation of the procedure time is another risk factor for RAO (15). However, a meta-analysis indicated that prolonged duration did not pose a risk for RAO, possibly as a result of the additional dose of heparin for the intervention (5). In our study, we suggested that a procedure time longer than 15 min had a predictive value for the development of RAO (OR: 1.03 CI: 1.01–1.05, *p*: 0.01).

It is known that heparin therapy is essential for the prevention of RAO. There was no difference in terms of RAO between the arterial or venous administration of heparin (5). Spaulding et al. reported the RAO rates as 70%, 24% and 4.3% in the groups that were administered without heparin, 2,000–3,000 IU heparin, and 5,000 IU heparin, respectively (16). In our study, we administered 5,000 IU of heparin to all the patients as a standard, and an additional dose of heparin (100 IU/kg) to the patients with prolonged processing time. The use of compression methods that allow distal blood flow after sheath removal at the end of the procedure has been found to be more advantageous in terms of RAO development than solid compression methods that do not allow distal blood flow (9). Tight aggressive prolonged compression leads to complete cessation of blood flow and eventual thrombus formation. TR band compression for two hours is indicated as negative predictive for RAO. In the study, we applied TR band compression in all the patients.

CHA2DS2-VASC scores were originally developed for stroke risk stratification in the patients with atrial fibrillation (17). However, many studies have shown that the CHA2DS2-VASC score may also be used in risk stratification of patients without atrial fibrillation (18,19). The CHA2DS2-VASc score has been studied extensively in terms of early and late complications in patients with acute coronary syndrome. In one study, it was determined that a CHA2DS2-VASc score of 3 or higher was associated with stent thrombosis. Studies have shown that CHA2DS2-VASc is associated with increased thrombotic and atherosclerotic processes in various cardiovascular diseases, regardless of the presence of atrial fibrillation (20). In this study, we showed that the cut-off value of ≥3 CHA2DS2-VASc score was predictive for RAO with sensitivity of 48.9% and specificity of 73.8% (OR: 1.44 CI 1.17–1.78 *P* < 0.001).

Following RAO, administering low molecular heparin (LMWH) for 2–4 weeks is currently the standard treatment. One study showed 31.5%–55.6% recanalization results in RAO patients treated with LMWH alone for 7 to 14 days (21). Similarly, another study reported that radial artery recanalization was 86.7% after four weeks of LMWH treatment (22). In our study, the recanalization rate after 2 weeks of DMHA treatment was found to be 66.6%. In addition, it was determined that the high CHA2DS2-VASc score was significantly associated with the persistence of arterial occlusion at the end of the two-week treatment (OR:1.37, 95% CI 1.01–1.85, *p*: 0.03). It may be said that high CHA2DS2-VASc score is a negative determinant of response to treatment after RAO.

The risk factors of the patient (sex, diabetes mellitus, arterial diameter, etc.), during the procedure (sheath size, heparinization, repetitive puncture), and after the application (tight compression) are effective in the development of RAO resulting from intimal damage and thrombotic event. Radial artery patency is important to prevent possible ischemia in the hand and for later surgical or interventional use. In patients with high risk factors, it is necessary to prevent the development of RAO and, if it develops, care should be taken in its treatment. The CHA2DS2-VASc score, which includes factors associated with thrombosis and atherosclerosis such as hypertension, diabetes mellitus, female sex, older age, and vascular disease, can provide valuable information in determining the risk of RAO and predicting the response to treatment after occlusion.

## Limitations

This study has several limitations. First of all, this is a single-center study. The results may not be generalized as the procedures were performed by a limited number of experts. All the patients were treated with a 6F sheath. We have not evaluated other sheath sizes. The percentage of RAO may differ in distinc dimensions. Another limitation is that we evaluated the transradial approach RAO rate one day later. There may be a late RAO, but we did not evaluate the late RAO. We did not assess the anticoagulation time in the diagnostic procedure after administering the cocktail solution. Finally, we administered two weeks of anticoagulation. We did not evaluate the patients for more than two weeks. The recanalization rate may be better after two weeks of treatment.

## Highlights

For the first time in the literature, we investigated and demonstrated that CHA2DS2-VASc score before coronary angiography may be related to the success of radial artery occlusion and recanalization. Although the relatively small number of patients, single-team experience might be considered among the study limitations, our results provide novel and practical findings which have clinical implications. Having a CHA2DS2-VASc score of ≥3, which can be easily applied in cardiology practice, has a predictive value for RAO. In addition, high CHA2DS2-VASc score has negative predictive value in evaluation response to treatment after occlusion. In conclusion, we observed that before coronary angiography CHA2DS2-VASc score was significantly and independently associated with success of recanalization as assessedwith acute radial trombozis patients regardless of AF presence who underwent treatment, which deserves to be verified with large scale prospective trials.

Generally, antiaggregant is not started in patients with normal coronary arteries unless there is a different antiaggregant indication. It should be kept in mind that the probability of radial artery thrombosis is high, even if the CHA2DS2-VASc score is high and the coronary arteries are found to be normal. We think that multiple punctures should be avoided in this patient group, TR band compression should be kept short if possible and antiaggregant treatment should be started for a short time.

## Data Availability

The original contributions presented in the study are included in the article, further inquiries can be directed to the corresponding author.
